# Executive Function Mediates the Relations between Parental Behaviors and Children's Early Academic Ability

**DOI:** 10.3389/fpsyg.2016.01902

**Published:** 2016-12-15

**Authors:** Rory T. Devine, Giacomo Bignardi, Claire Hughes

**Affiliations:** Centre for Family Research, Department of Psychology, University of CambridgeCambridge, UK

**Keywords:** executive function, academic ability, parenting, scaffolding, longitudinal study

## Abstract

The past decade has witnessed a growth of interest in parental influences on individual differences in children's executive function (EF) on the one hand and in the academic consequences of variation in children's EF on the other hand. The primary aim of this longitudinal study was to examine whether children's EF mediated the relation between three distinct aspects of parental behavior (i.e., parental scaffolding, negative parent-child interactions, and the provision of informal learning opportunities) and children's academic ability (as measured by standard tests of literacy and numeracy skills). Data were collected from 117 parent-child dyads (60 boys) at two time points ~1 year apart (M Age at Time 1 = 3.94 years, *SD* = 0.53; M Age at Time 2 = 5.11 years, *SD* = 0.54). At both time points children completed a battery of tasks designed to measure general cognitive ability (e.g., non-verbal reasoning) and EF (e.g., inhibition, cognitive flexibility, working memory). Our models revealed that children's EF (but not general cognitive ability) mediated the relations between parental scaffolding and negative parent-child interactions and children's early academic ability. In contrast, parental provision of opportunities for learning in the home environment was directly related to children's academic abilities. These results suggest that parental scaffolding and negative parent-child interactions influence children's academic ability by shaping children's emerging EF.

## Introduction

Meta-analytic evidence from longitudinal research demonstrates that early academic abilities, such as a rudimentary understanding of mathematics and basic literacy, provide an important foundation for later academic achievement (e.g., Duncan et al., 2007). Attempts to understand the sources of individual differences in these foundational abilities have generated a substantial body of developmental research such that extensive data is now available on the relations between early language skills, general intelligence, and rudimentary academic skills (e.g., La Paro and Pianta, 2000; Roth et al., 2015). In parallel, recent decades have seen a growth of interest in how children's early academic abilities relate to parental behaviors, on the one hand, and children's emerging executive functions (EF–the suite of cognitive processes involved in the control of thoughts and actions) (Blair and Raver, 2015) on the other hand. Integrating these twin strands of research, the present study sought to examine whether variation in children's EF might play a mediating role in the association between preschool parent-child interactions and early academic ability.

## Parental influences on children's academic ability

Variation in children's early academic ability is linked to both domain-general parental influences (e.g., the emotional quality and level of cognitive support that parents provide) and domain-specific parental influences (e.g., activities targeted at literacy and numeracy) (e.g., Kluczniok et al., 2013). Perhaps one of the most widely-studied of these different parental influences on children's academic ability has been the home learning environment (HLE), a term that refers to the extent to which resources and informal learning opportunities are available in the home. Children's HLEs are often studied using interviews and observer ratings of the home environment, such as the Home Observation for Measurement of the Environment (HOME) (Bradley et al., 2003; Totsika and Sylva, 2004) and, more recently, self-report questionnaires (Melhuish et al., 2008). Pioneering longitudinal studies demonstrated that aspects of the HLE (such as the provision of structured activities) were positively related to cognitive development in the early years (Bradley et al., 1979). Follow-up studies revealed that children's HLE at age 2 predicted children's academic performance in reading and languages at age 10 (Bradley et al., 1988). Subsequent studies have demonstrated that the HLE is positively related to children's language skills (e.g., Son and Morrison, 2010), literacy (e.g., Hindman and Morrison, 2012), and adjustment in the classroom (e.g., Lamb Parker et al., 1999). Longitudinal evidence also shows that children's HLE in the preschool years is positively correlated with mathematics ability in early and middle childhood (Melhuish et al., 2008).

Alongside the availability of informal learning opportunities in the home, researchers have examined how the quality of parent-child interactions, through specific attempts to provide cognitive support, might foster children's academic ability. In seminal work that applied socio-cultural theories of cognitive development (Vygotsky, 1978) to understand the contribution of parental tutoring practices, Wood et al. (1976) argued that parents (or other skilled adults or peers) who tailor their support can “scaffold” children's ability to solve problems independently (Wood and Wood, 1996). The most effective way to do this was through use of the “contingency rule” (Wood and Wood, 1996). That is, when children struggle to complete a task parents should increase the level of support they provide and when children succeed parents should decrease the level of support they provide (Wood and Wood, 1996). Parents' use of the contingency rule is typically measured through detailed observations of sequences of task-related behavior during parent-child interactions (e.g., Meins, 1997; Carr and Pike, 2012). Since the late 1980s, studies of the correlates and consequences of variation in parental use of the contingency rule have shown associations with children's success both on the shared task (Pratt et al., 1988) and on related tasks completed independently (Conner et al., 1997). Crucially, the effects of parents' use of the contingency rule appear to extend beyond the immediate task context. Cross-sectional studies show that parental use of the contingency rule is related, in early childhood, to children's observed persistence, self-control and help-seeking behavior in the classroom (Neitzel and Stright, 2003) and, in middle childhood, to children's mathematics performance (Pratt et al., 1992) and teacher-rated academic competence (Mattanah et al., 2005). These findings suggest that parental use of the contingency rule during problem-solving tasks might benefit children's academic ability. However, longitudinal relations with measures of academic ability in early childhood have yet to be examined.

Alongside parents' cognitive support, global measures of the affective quality (e.g., warmth, positivity, responsiveness) of parent-child interactions appear positively related to: (i) preschool children's early academic skills (as measured by tests of language ability and parent-rated school-readiness) (Leerkes et al., 2011); (ii) literacy, mathematics and teacher-rated academic competence in middle childhood (e.g., NICHD Early Child Care Research Network, 2008); and (iii) academic achievement in adolescence (Jimerson et al., 2000). Conversely, negative parent-child interactions characterized by harshness, negative control, and negative affect are associated with teacher-ratings of poor academic adjustment (e.g., Pettit et al., 1997; Culp et al., 2000) and poor performance on standard tests of achievement in middle childhood (Harold et al., 2007).

As outlined above, there is good evidence that individual differences in children's academic abilities are associated with a variety of measures of the family environment including the quantity and quality of cognitive support on the one hand and the affective quality of interactions on the other. What is not yet understood, however, is what mechanisms underpin these associations. At least three different pathways between these distinct aspects of parental behavior and variation in children's early academic ability deserve note. First, the HLE might be related to early academic ability for the simple reason that frequent exposure to basic literacy and numeracy activities provides children with opportunities to practice in these domains (e.g., Kluczniok et al., 2013). Second, with regard to the relations between parental use of the contingency rule and children's academic ability, it is conceivable that parents who provide appropriate support continually challenge their children's nascent cognitive abilities it is conceivable that parents who provide appropriate support continually challenge their children's nascent cognitive abilities. Third, turning to the affective quality of parent-child interactions, Blair and Raver (2015) have proposed a psychobiological framework that emphasizes the interplay between stress, early cognition, and academic ability. According to this account stress physiology mediates the impact of early stressful experiences (such as negative parent-child interactions) on cognitive development and early academic ability (Blair et al., 2011). While these three pathways may each exert a specific influence on distinct aspects of children's developing cognition, they are not mutually exclusive and may operate in concert. Indeed, existing studies either aggregate these different aspects of parental behavior or focus on a single measure of parental behavior. One drawback of this approach is that both the intervening processes and the relative salience of each of these measures in predicting children's academic abilities remain poorly understood. Addressing these gaps, a key goal of the present study was to elucidate the mechanisms by which parental behaviors relate to individual differences in children's academic ability.

## Executive function, academic ability, and parental influences

One way to understand better the relations between parental behaviors and children's academic ability is to extend the focus of research onto other, more fundamental, cognitive abilities that are related to both children's academic ability and parental behavior. Research interest in the relations between children's executive function (EF) and academic abilities has grown dramatically in recent years (e.g., Blair and Raver, 2015; Ursache et al., 2012). EF encompasses skills such as over-riding entrenched habitual responses (or “inhibition”), updating information held in mind (or “working memory”) and switching between tasks (or “cognitive flexibility”) (e.g., Diamond, 2013). In adolescence and adulthood, studies of the structure of EF support a “unity and diversity” model. That is, each aspect of EF is comprised of variance that is specific to that component of EF and variance that overlaps with other aspects of EF producing distinct but correlated factors representing inhibition, working memory and flexibility (Miyake and Friedman, 2012). In early childhood however, EF studies support a “unity” model in which a single latent EF factor explains individual differences in performance across a diverse range of tasks (e.g., Wiebe et al., 2008).

A substantial body of evidence shows that there are significant associations between diverse measures of EF and objective tests of mathematics and literacy in early and middle childhood (e.g., Willoughby et al., 2012). EF makes a unique contribution to academic ability above and beyond language ability or general cognitive ability indicating that correlations between EF and academic ability cannot be explained by these factors (e.g., Espy et al., 2004; Blair and Razza, 2007). Moreover, longitudinal studies also demonstrate that EF in early childhood predicts later academic ability even when earlier measures of academic performance are taken into account suggesting that EF is linked to *gains* in academic ability (e.g., Clark et al., 2013; Fuhs et al., 2014; Nesbitt et al., 2015). Underscoring this point, intervention studies indicate that gains in EF result in improved academic abilities, suggesting causal relations between these variables in early childhood (e.g., Raver et al., 2011).

Mirroring the growing interest in parental influences on children's academic ability, researchers have also begun to elucidate the ways in which early family experiences shape children's EF (e.g., Müller et al., 2013). Just as academic ability has been linked to the quality and quantity of cognitive and emotional support that parents provide, the development of EF has also been studied in relation to a range of parental behaviors. Factors such as household routines and chaotic family environments show concurrent and longitudinal negative associations with EF in early childhood (e.g., Hughes and Ensor, 2009; Vernon-Feagans et al., 2016). Early literacy and numeracy activities place considerable demands on children's EF (Blair and Raver, 2015). For example, reading activities require children to shift their attention between phonemes and whole words (Blair and Raver, 2015). It is therefore conceivable that through frequent exposure to informal literacy and numeracy activities in the home, the HLE might be correlated with individual differences in EF.

At the level of parent-child interactions, cognitive aspects of parent-child interactions such as parental verbal scaffolding during problem-solving tasks in early childhood show both concurrent and longitudinal associations with EF in early childhood (Hughes and Ensor, 2009; Bernier et al., 2010; Hammond et al., 2012). There is also evidence that the affective quality of parent-child interactions in early childhood is related to children's EF. There are moderate concurrent and longitudinal associations between maternal depression and variation in children's EF in early childhood indicating that exposure to negative parental affect may adversely affect children's early cognitive development (e.g., Blair et al., 2011; Hughes et al., 2013). Crucially, both cognitive and affective dimensions of parental behavior show unique associations with EF that are independent of children's language ability or, in the case of longitudinal studies, children's earlier performance on measures of EF (e.g., Hughes and Ensor, 2009).

## Does EF mediate the relation between parental behavior and academic ability?

**Figure 1:**
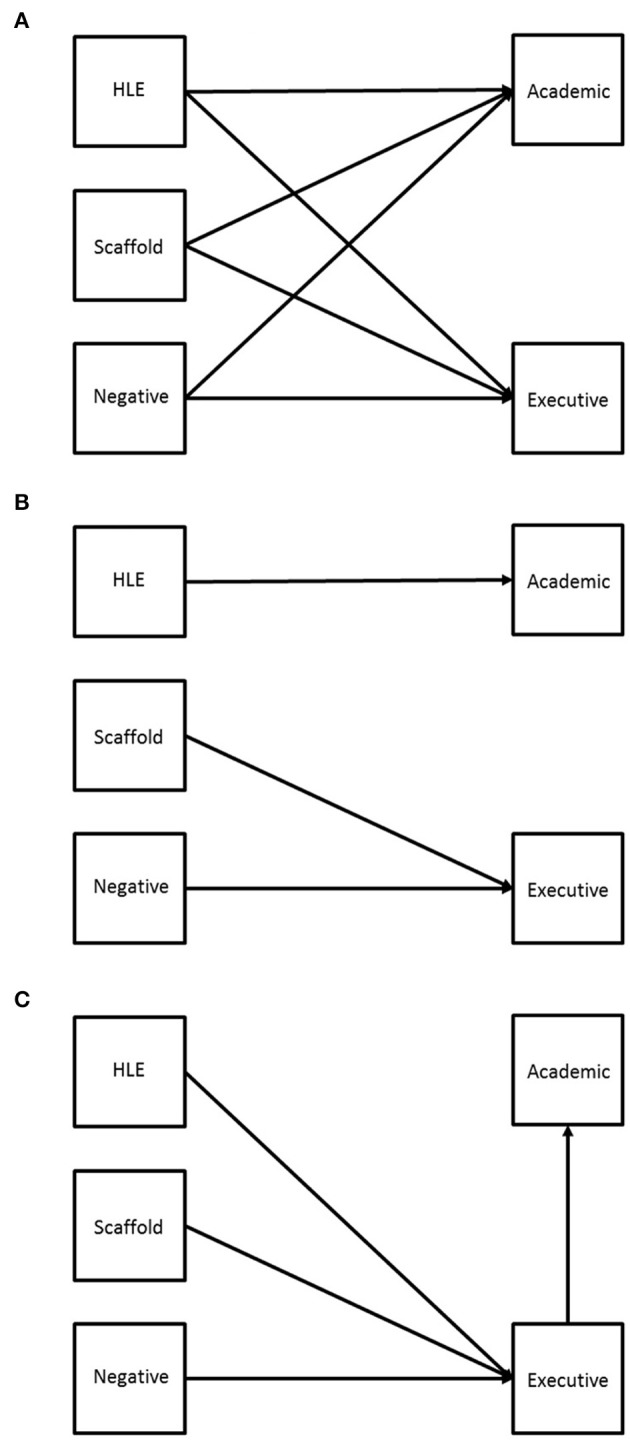
**Theoretical models linking parental behaviors and child outcomes**. Parental Behaviors are depicted on the left hand side of each model and child outcomes are depicted on the right hand side of each model. HLE, Home Learning Environment; Scaffold, Contingent Scaffolding Behavior; Negative, Negative Parent-Child Interactions. **(A)** Model 1. Domain General Model; **(B)** Model 2. Domain Specific Model; **(C)** Model 3. Mediation Model.

One interpretation of the common associations between parental behavior and both EF and children's academic ability is that the quantity and quality of parental cognitive support and/or the affective quality of parent-child interactions could foster cognitive development in a range of domains (e.g., EF, early literacy and math ability). According to this Domain General Model, high levels of parental cognitive support and low levels of negative parent-child interactions might combine to exert a general influence on children's cognitive development (see Figure 1A). Alternatively, according to the Domain Specific Model, different aspects of parental behavior may show specific associations with distinct aspects of children's cognitive abilities. For example, the HLE might show direct associations with children's academic ability while negative parent-child interactions might show unique associations with children's EF (see Figure 1B). Another possibility is that child EF may play a mediating role in the associations between different dimensions of parental behavior and children's academic ability (see Figure 1C). Indirect support for this Mediation Model comes from two reports based on data from the NICHD Study of Early Child Care that have measured constructs that are closely related to core domains of EF.

First, children's sustained attention and impulsivity at age 4.5 years partially mediated the relation between parenting quality (as measured by a composite index of physical and social resources in the home, observer ratings of parental sensitivity and cognitive stimulation) at 4.5 years and children's academic achievement (as measured by performance on standardized reading and mathematics tests) at age 6 (NICHD Early Child Care Research Network, 2003). Second, in the same sample, children's performance on a test of planning ability (considered to assess multiple aspects of EF including inhibition, working memory and flexibility—Russell, 1996) at ages 6 and 8 mediated the relations between parenting quality at 4.5 years and children's later academic ability at 8 and 10 years by Friedman et al. (2014). Alongside these results, Fitzpatrick et al. (2014) found that more traditional measures of EF partially mediated the relation between socio-economic status (SES) and children's academic ability in a sample of children aged between 3 and 5 years of age. Together these findings suggest that aspects of children's home environments might encourage the development of EF which in turn enhances children's early academic abilities. That said, the available evidence does not specify which aspects of parental behavior (i.e., cognitive or affective) matter most for academic achievement. Moreover, it is unclear from existing work whether EF in particular (rather than general cognitive ability) accounts for the relations between parental behavior and children's academic ability.

## Summary of aims

The present longitudinal study had two primary aims. First, we sought to examine the independence and overlap in the relations between measures of parental behavior (i.e., the home learning environment, negative parent-child interactions, and parental scaffolding) and children's early academic ability. Our second aim was to examine the relations between parental behavior, children's EF and academic ability by testing the direct and indirect relations between these constructs (as shown in Figure 1). In each of our analyses we sought to examine the unique effects of parental behaviors on children's academic ability by controlling for individual differences in known correlates of academic ability such as early measures of verbal ability, general cognitive ability, and parental education.

## Methods

### Participants

Parents and children were recruited from nurseries, shopping centers and playgroups in the East of England. To be included in the study children had to be aged either 3 or 4 years old, be native English speakers and have no reported history of developmental delay. One hundred twenty parent-child dyads took part in the first wave of laboratory visits (Time 1). Of this group 117 dyads (60 boys) agreed to be contacted for a follow-up study. Although socio-economically homogenous (81% of parents had completed an undergraduate degree), the sample were ethnically diverse (66% White British). Of these 117 families, 100% of the families were contacted at the second wave of visits. Two families were no longer eligible to participate as they had left the country. Of the eligible 115 families 103 (90%) completed the second visit (Time 2) approximately 13 months later, *SD* = 1.65 months, range: 11–17 months. The average age of children was 3.94 years, *SD* = 0.53, range: 3–4.95 years, and 5.11, *SD* = 0.54, range: 4–6.10 years, at Time 1 and 2 respectively. Binary logistic regression revealed that although non-returners did not differ from those who returned for the second visit in age, gender, or general cognitive ability (as measured by the Object Assembly task), non-returners were marginally more likely to have low levels of parental education, OR = 3.05, *B* = 1.12, *SE* = 0.64, *p* = 0.08.

### Procedures

All procedures were approved by the local University Research Ethics Committee. Parents and children were invited to participate in two laboratory visits lasting up to 75 min in length (including time for information and consent, rest breaks and debriefing) approximately 1 year apart. Following written parental consent, children completed a battery of tasks designed to measure EF, general cognitive ability and early academic ability. Individual child testing lasted approximately 30 min. The children completed the task battery in a fixed counter-balanced format such that no two tasks from any domain were completed alongside one another. Children were provided with rest breaks and rewarded with stickers for the completion of each task. Parents completed a short questionnaire booklet in an adjoining room while children completed the task battery. Upon completion of cognitive testing, parents were observed interacting with their child during 5 min of structured play with a set of jigsaw puzzles. At the end of each session parents were debriefed and provided with £15 and a small gift for their child.

### Measures

#### Early academic ability

Children completed two subtests from the Wechsler Individual Achievement Test (WIAT-II-UK) (Rust and Golombok, 2005) at Time 2 to provide a measure of early academic ability. The *Word Reading* subtest was designed to measure a range of early reading skills including phonological awareness, letter–sound awareness, and letter reading skills. The *Mathematics Reasoning* subtest was designed to measure children's ability to count, identify numbers and shapes and solve simple mathematical problems. For both tasks the items were presented on a color flipbook and 1 point was awarded for each correctly answered question. Children completed up to 47 items on the Word Reading subtest and up to 35 items on the Mathematics Reasoning subtest. Scores on the two WIAT-II-UK subtests were strongly correlated, *r*_(101)_ = 0.73, *p* < 0.001, and so were standardized and averaged to create a single “Academic Ability” variable (α = 0.85).

#### Executive function

Children completed a short battery of tasks designed to measure EF at Time 1 and 2. To index conflict inhibition the children completed the *Happy/Sad Task* (Lagattuta et al., 2011) at both time points. In this task children were shown two cards depicting either a yellow “happy face”or a yellow “sad face.” First the children were asked to point to the happy face and then to the sad face. Following this the experimenter told the children that they would play a “silly game” so that when the experimenter said “happy” the child had to point to the sad face and when the experimenter said “sad” the child had to point to the happy face. The children received 4 training trials with feedback from the examiner. If the child made an error on one of these training trails, up to two further sets of 4 training trials were provided and the rules were re-stated. If children failed these training trials they were assigned a score of 0 and testing was discontinued. Children completed 20 test trials in a fixed order with no feedback. The total number of correct items was summed together.

Children also completed the *Dimension Change Card Sort* (DCCS) Task (Zelazo, 2006) at both time points. This task was designed to measure children's ability to switch between rules and administered according to Zelazo's (2006) protocol. The children completed the pre-switch and post-switch phases at Time 1 and 2 and the border game at Time 2 only. In each phase the children were shown two laminated cards (one depicting a blue rabbit and the other depicting a red boat) attached to two sorting boxes and were required to sort six cards depicting either a blue boat or red rabbit. Following a demonstration of how the cards should be sorted the children completed either six trials of the “color game” or six trials of the “shape game” (counter-balanced across participants). In the color game the children had to place up to three cards depicting the red rabbit next to the target card showing the red boat and up to three cards showing the blue boat next to the target card showing the blue rabbit. In the shape game the children had to place the sorting cards depicting the red rabbit next to the blue rabbit target card and the cards depicting the blue boat next to the red boat target card. This first game served as the pre-switch phase. All children passed the pre-switch phase (i.e., sorted 5 or more cards correctly). Following the pre-switch phase, the children playing the color game proceeded to the shape game and *vice versa*. Before this post-switch phase the children were told that the rules had changed (and the new rule was repeated before each sort). Children were awarded 1 point for each correctly sorted card in the post-switch phase. At Time 2 those children who passed the post-switch phase (i.e., sorted 5 or more cards correctly) proceeded to the border game. In the border game the children completed a further 12 sorting trials using a third set of cards containing 6 normal sorting cards and 6 cards with a thick black border. Cards without a border were sorted according to one rule (e.g., shape game) and cards with a black border were sorted according to another rule (e.g., color game). Children were awarded 1 point for each correctly sorted card. Children who failed the post-switch phase were scored 0 on the border game.

To measure working memory the children completed the *Self-Ordered Pointing Task* (SOPT) (Cragg and Nation, 2007) at both time points. In this task the children were shown a color flipbook depicting an increasing number of colored pictures of single syllable concrete objects (ranging from 2 objects to 7 objects with two sets in each number) in one of 16 locations on the page. In each set care was taken to ensure that no two objects were taken from the same class of objects (e.g., fruits, toys, pets). Children were asked to point to a new picture on each page and were told that they could not select the same picture twice. For example the first page depicted two images (e.g., bowl, flag) and the second page depicted the same images but in different spatial locations. The number of repetition errors (i.e., repeated points to the same picture) were recorded and used as an index of working memory. These error scores were reflected to be consistent with the other EF measures.

At Time 2 the children also completed the *Day/Night Task* (Gerstadt et al., 1994) to measure conflict inhibition. This task was not completed at Time 1 because during pilot testing we found that the youngest children in our sample became too fatigued. The Day/Night task followed the same general procedure as the Happy/Sad task but instead of cards depicting happy and sad faces, the experimenter presented the children with two laminated cards depicting either the sun or the moon. Children were required to point to the picture of the sun when the experimenter said “night” and to the picture of the moon when the experimenter said “day.” The children completed 20 trials and were awarded 1 point for each correct trial.

#### General cognitive ability and verbal ability

Children completed three subtests from the Wechsler Preschool and Primary Scale of Intelligence (WPPSI-III-UK) (Rust, 2003). To obtain an index of general cognitive ability the children completed the *Object Assembly* task at Time 1. In this task participants were required to assemble a set of puzzles showing cartoon images of objects (e.g., clock, bird, hotdog). Children received marks for each correctly aligned juncture in the first 90 s of each trial. The children completed up to 14 trials and the scores from each trial were summed together. At Time 2 children completed the *Matrix Reasoning* task. In this task the children had to complete a matrix by identifying the missing portion from a choice of 4 or 5 options presented in a color flipbook. Children completed up to 29 trails and scores from each trial were summed together. The Matrix Reasoning task could not be used at Time 1 as it is only suitable for use with children aged over 4 years (Rust, 2003). To measure verbal ability the children completed the *Receptive Vocabulary* task at Time 1. The children were shown a color flipbook depicting 4 images on each page and asked to point to the picture that matched the word uttered by the experimenter. Children completed up to 38 trials and were awarded 1 point for each correctly identified picture.

#### Parental behavior

At Time 1 parents and children were recorded for 5 min playing together using wall-mounted unobtrusive digital cameras while the experimenters were in another room. Parents and children were provided with three jigsaw puzzles (a 6, 8, and 12 piece puzzle) from the Galt Velvet Puzzles Jigsaw set. The parents were instructed to work together with their child to complete as many of the three puzzles as possible within 5 min. The data from these videos were then coded off-line using two different coding schemes by different trained researchers naive to the participants' identities and test scores.

*Negative Parent-Child Interaction* was measured using items from the Parent-Child Interaction System (PARCHISY) coding scheme (Deater-Deckard et al., 1997). Raters scored parental behavior during the task on three 7-point rating scales (ranging from “none” to “exclusive/constant”): Negative control (i.e., use of physical control, use of criticism), negative affect (i.e., frowning, harsh tone of voice) and conflict (i.e., disagreement, arguing or tussling). Following training from an experienced rater 25 video clips were randomly selected for double coding. Intra-class correlations for each item were acceptable: Negative content, ICC = 0.89, negative affect, ICC = 0.75, and conflict, ICC = 0.74. The remaining clips were double-coded and scores were averaged across raters.

*Parental Scaffolding* was measured using a coding scheme developed by Wood and Middleton (1975) and refined by Meins (1997). This approach required coding each of the verbal and non-verbal task-related behaviors of parents and children during the 5-min observation. Parental interventions were assigned into one of five mutually exclusive categories ranging from more open-ended verbal suggestions to more specific physical demonstrations: Level 1 Orienting Verbal Suggestions (e.g., “Let's start with the corners”); Level 2 Suggestions about Specific Pieces or Locations or Actions (e.g., “Try turn that piece around”); Level 3 Verbal Solutions (e.g., “This piece goes here”); Level 4 Direct Physical Solutions (e.g., Caregiver hands child a piece for a specific location); Level 5 Physical Demonstrations (e.g., Caregiver assembles or dismantles parts of the puzzle). Children's responses were coded as either “success” (i.e., correct placement of the puzzle piece) or “failure” (i.e., incorrect placement of the piece). Following training, 25 clips were randomly selected and double coded. ICCs were acceptable for all codes: Level 1 interventions, ICC = 0.64, Level 2 interventions, ICC = 0.85, Level 3 interventions, ICC = 0.97, Level 4 interventions, ICC = 0.98, Level 5 interventions, ICC = 0.96, frequency of child successes, ICC = 0.99, and frequency of child failures, ICC = 0.94.

The sequences of parent-child codes were parsed into three-turn chains of parent interventions, child actions and parent responses. If multiple interventions preceded a child action only the highest level of intervention was selected (Wood and Middleton, 1975; Carr and Pike, 2012). These three-turn chains were used to analyse the contingency between parents and children during the task. We tallied the number of times that parents shifted “up” (i.e., moving from a less specific to more directive intervention level), shifted “down” (i.e., moving from directive to less specific intervention level) and remained at the same level of intervention (“no shift”) after each child success and failure. Variation in parental scaffolding reflected parents' use of the contingency rule (Wood and Middleton, 1975; Meins, 1997; Carr and Pike, 2012), that is, the successful placement of a piece by a child should be followed by an intervention at the same or at a lower level of specificity and failure to place a piece correctly should be followed by an intervention that is one or two levels higher than the previous level of intervention. Contingency or “scaffolding” scores were calculated by summing the total number of times that parents shifted appropriately after success or failure and dividing this by the total number of parental interventions after each success or failure. Scores ranged from 0 (no evidence) to 1 (exclusive use of the contingency rule).

The *Home Learning Environment* (HLE) was measured at Time 2 using the Home Learning Environment Index (Melhuish et al., 2008). This seven item self-report questionnaire records the frequency with which parents and children engage in informal learning activities. Parents were asked whether or not they engaged in seven activities with their children (e.g., reading at home, teaching numbers, and counting) and then how often the engaged in each activity on a 7-point scale (ranging from “occasionally or less than once a week” to “7 times a week/constantly”). Parents indicating that they did not engage in the learning activity with their child received a score of 0 for that item. The internal consistency of the measure was acceptable (α = 0.73) and so the scores from each item were summed together. While there was insufficient time to administer this test at Time 1, longitudinal findings demonstrate that individual differences on measures of the HLE show remarkable stability across early childhood (e.g., Lehrl et al., 2016).

## Results

### Analytic strategy

We conducted our primary data analyses using *MPlu*s Version 7 (Muthén and Muthén, 2012) using a robust maximum likelihood estimator which is suitable for non-normally distributed data and small sample sizes (Brown, 2015). For each of the 103 participants returning there were no missing data points for EF, general cognitive ability or academic ability at Time 2. To avoid loss of data we used a full information approach to analyzing the data so that all cases (*N* = 117) with data at Time 1 could be included in the analyses. Missing values were estimated in *MPlu*s using the robust maximum likelihood estimator (Muthén and Muthén, 2012). *MPlu*s does not impute data but instead estimates missing model parameters and standard errors using all of the available data (Enders, 2001; Asparouhov and Muthén, 2010). The full information approach can be used in regression models and is preferable to traditional approaches to handling missing data (e.g., list-wise deletion, mean substitution) because it produces less biased estimates and does not require that data are missing completely at random (i.e., missingness is unrelated to any other variable in the dataset or performance on the variable itself) (Enders, 2001; Acock, 2005).

Since the WIAT-II-UK was not age-appropriate for all the children at Time 1 we controlled for individual differences in early cognitive ability by regressing academic ability scores onto earlier measures of verbal ability, general cognitive ability (as measured by the Object Assembly task and Matrix Reasoning task) and EF as well as concurrent age. Structural equation modeling in *MPlus* allowed us to examine simultaneously the direct and indirect effects (via EF and general cognitive ability at Time 2) of each of the parental variables on academic ability (Cole and Maxwell, 2003; Preacher, 2015). We have provided a more detailed analysis of the longitudinal relations between parental behaviors and children's EF elsewhere (Hughes and Devine, under review). We evaluated the fit of our models using Brown's (2015) four criteria: A non-significant χ^2^ test of model fit, comparative fit index (CFI) ≥ 0.95, Tucker Lewis index (TLI) ≥ 0.95, and root mean square error of approximation (RMSEA) ≤ 0.08. We evaluated the strength of correlations using Cohen's (1988) criteria: Small/weak (0.10), medium/moderate (0.30), and large/strong (0.50).

### Descriptive statistics and data reduction

Table 1 presents descriptive statistics for the key study variables. Our first step was to create composite scores in order to increase reliability (Rushton et al., 1983) and simplify our analyses. We conducted a series of CFAs to inform the creation of composite scores for different variables in our study. Each of the PARCHISY items were significantly inter-correlated, 0.36 < *r* < 0.54, all *p*s < 0.01. We tested a one-factor model in which each of the PARCHISY items loaded onto a single “negative parent-child interaction” latent factor. This model was “just-identified” (i.e., there were an equal number of model parameters and variances/co-variances in the sample matrix) and while model fit indices could not be calculated, parameter estimates could still be calculated and interpreted (Brown, 2015). We set the metric of the latent factor by fixing the loading of the first indicator to 1. The latent factor exhibited significant variance, unstandardized estimate = 0.45, *p* = 0.007. All item loadings were significant; Conflict Standardized Estimate = 0.80, *p* < 0.001, Negative Affect Standardized Estimate = 0.68, *p* < 0.001, Negative Control Standardized Estimate = 0.52, *p* < 0.001. Factor determinacy co-efficient values range from 0 to 1 and higher values (≥0.80) indicate higher internal consistency (Brown, 2015). The negative parent-child interaction latent factor had a factor determinacy co-efficient of 0.87.

**Table 1:** **Descriptive statistics**.

	**Time 1**			**Time 2**		
	**M (*SD*)**	**95% CI**	**Range**	**M (*SD*)**	**95% CI**	**Range**
**CHILD MEASURES**						
Happy-sad task	13.35 (6.30)	12.17, 14.53	0–20	17.24 (2.42)	16.77, 17.71	7–20
DCCS post-switch	3.74 (2.50)	3.29, 4.19	0–6	5.36 (1.63)	5.04, 5.67	0–6
Self-ordered pointing task	0.13 (0.06)	0.12, 0.14	0–0.28	0.08 (0.04)	0.07, 0.09	0–0.22
DCCS border game	–	–	–	6.57 (2.86)	6.02, 7.12	0–12
Day-night task	–	–	–	17.26 (1.98)	16.87, 17.65	12–20
Receptive vocabulary	23.04 (5.12)	22.12, 23.96	8–31	–	–	–
Object assembly T1/matrix task T2	16.71 (8.24)	15.22, 18.20	2–35	11.73 (4.84)	10.79, 12.66	4–24
WIAT word	–	–	–	32.14 (13.82)	29.47, 34.81	2–46
WIAT mathematics	–	–	–	15.30 (5.17)	14.30, 16.30	5–28
Academic ability	–	–	–	50.00 (9.30)	48.20, 51.80	30.54–66.57
Executive function	49.99 (7.72)	48.59, 51.38	32.16–62.66	50.01 (7.01)	48.66, 51.36	28.17–63.66
**PARENT MEASURES**						
Negative parent-child interaction	49.94 (7.89)	48.44, 51.44	41.26–82.49	–	–	–
Parental use of contingency rule	0.42 (0.20)	0.38, 0.46	0–1	–	–	–
Home learning environment	–	–	–	30.44 (9.78)	28.56, 32.32	8–49

Consistent with previous studies (e.g., Hughes and Ensor, 2005) the correlations between measures of EF were moderate at Time 1, 0.29 < *r* < 0.48, Mean *r* = 0.40, and weak to moderate at Time 2, 0.08 < *r* < 0.73, Mean *r* = 0.33. The SOPT error score at Time 2 was not correlated with any other measure of EF at Time 2 and so was not included in any further analyses. Drawing on the “unity” model of individual differences in EF (described earlier), we tested a model in which each of the three EF indicators at Time 1 loaded onto a one latent factor and each of the four EF indicators at Time 2 loaded onto another latent factor. The error terms for the two DCCS indicators at Time 2 were permitted to correlate. This model provided an acceptable fit to the data, $\chi_{\left(12\right)}^{2}$ = 16.47, *p* = 0.17, CFI = 0.97, TLI = 0.96, RMSEA = 0.06. All indicators loaded significantly onto the Time 1 EF latent factor; Happy/Sad Task Standardized Estimate = 0.78, *p* < 0.001, DCCS Standardized Estimate = 0.61, *p* < 0.001, SOPT Standardized Estimate = 0.50, *p* < 0.001. All but one of the Time 2 indicators loaded significantly onto the Time 2 EF latent factor; Happy/Sad Task Standardized Estimate = 0.67, *p* < 0.001, DCCS Border Game Standardized Estimate = 0.38, *p* < 0.01, DCCS Standardized Estimate = 0.24, *p* = 0.07, Day/Night Task Standardized Estimate = 0.60, *p* < 0.001. Both latent factors exhibited significant variance at Time 1, Unstandardized Estimate = 23.73, *p* < 0.001, and at Time 2, Unstandardized Estimate = 2.60, *p* < 0.01. The factor determinacy co-efficient was 0.87 for the Time 1 latent factor and 0.84 for the Time 2 latent factor.

### Relations between parental behavior, EF, and academic ability

Table 2 shows the sample correlations between each measure of parental behavior. These show that negative parent-child interaction was weakly positively correlated with the HLE. Parental scaffolding and the HLE were unrelated. Each parental measure showed weak correlations with academic ability in the expected directions. We calculated partial correlations controlling for individual differences in age and general cognitive ability (as measured by the Matrix Reasoning task) at Time 2. Academic ability was weakly correlated with each aspect of parental behavior: Negative parent-child interaction, *pr*_(100)_ = −0.19, *p* = 0.05; parental scaffolding, *pr*_(100)_ = 0.17, *p* = 0.09; the HLE, *pr*_(100)_ = 0.27, *p* = 0.005. Table 2 also shows the correlations between each measure of parental behavior and individual differences in EF at Time 2. Once again we examined these relations further using partial correlations controlling for individual differences in age at Time 2. EF remained significantly correlated with both negative parent-child interaction, *pr*_(100)_ = −0.29, *p* = 0.003, and parental scaffolding, *pr*_(100)_ = 0.29, *p* = 0.003, but showed a weak and non-significant correlation with the HLE, *pr*_(100)_ = 0.13, *p* = 0.19.

**Table 2:** **Sample correlations for key study variables**.

	**1**	**2**	**3**	**4**	**5**	**6**	**7**	**8**	**9**	**10**	**11**	**12**
1. Academic ability T2	−											
2. Executive function T1	0.64^**^	−										
3. Executive function T2	0.68^**^	0.49^**^	−									
4. Object assembly T1	0.52^**^	0.43^**^	0.42^**^	−								
5. Matrix reasoning T2	0.54^**^	0.31^**^	0.39^**^	0.42^**^	−							
6. Receptive vocab. T1	0.44^**^	0.37^**^	0.42^**^	0.39^**^	0.37^**^	−						
7. Negative interaction T1	−0.19^+^	−0.09	−0.29^**^	−0.25^**^	−0.12	−0.21^**^	−					
8. Contingency rule T1	0.13	0.18^+^	0.26^**^	0.02	−0.08	0.10	−0.12	−				
9. HLE T2	0.20^*^	0.03	0.12	−0.04	0.17^+^	0.06	0.17^+^	0.05	−			
10. Age (concurrent)	0.73^**^	0.55^**^	0.51^**^	0.52^**^	0.48^**^	0.52^**^	−0.06	0.06	0.01	−		
11. Gender	−0.11	−0.09	−0.15	0.10	0.02	−0.16^*^	0.19^+^	−0.06	−0.25^**^	0.05	−	
12. Parental education	0.14	0.16	0.15	0.12	0.19^+^	0.15	0.05	0.20^*^	0.05	−0.01	0.06	−

** p < 0.01.

* p < 0.05.

+ *p < 0.10. Vocab, Vocabulary; Negative Interaction, Negative Parent-Child Interaction; Contingency Rule, Parental use of Contingency Rule; HLE, Home Learning Environment; T1, Time 1; T2, Time 2*.

### Direct and indirect effects of parental behavior on academic ability

**Figure 2:**
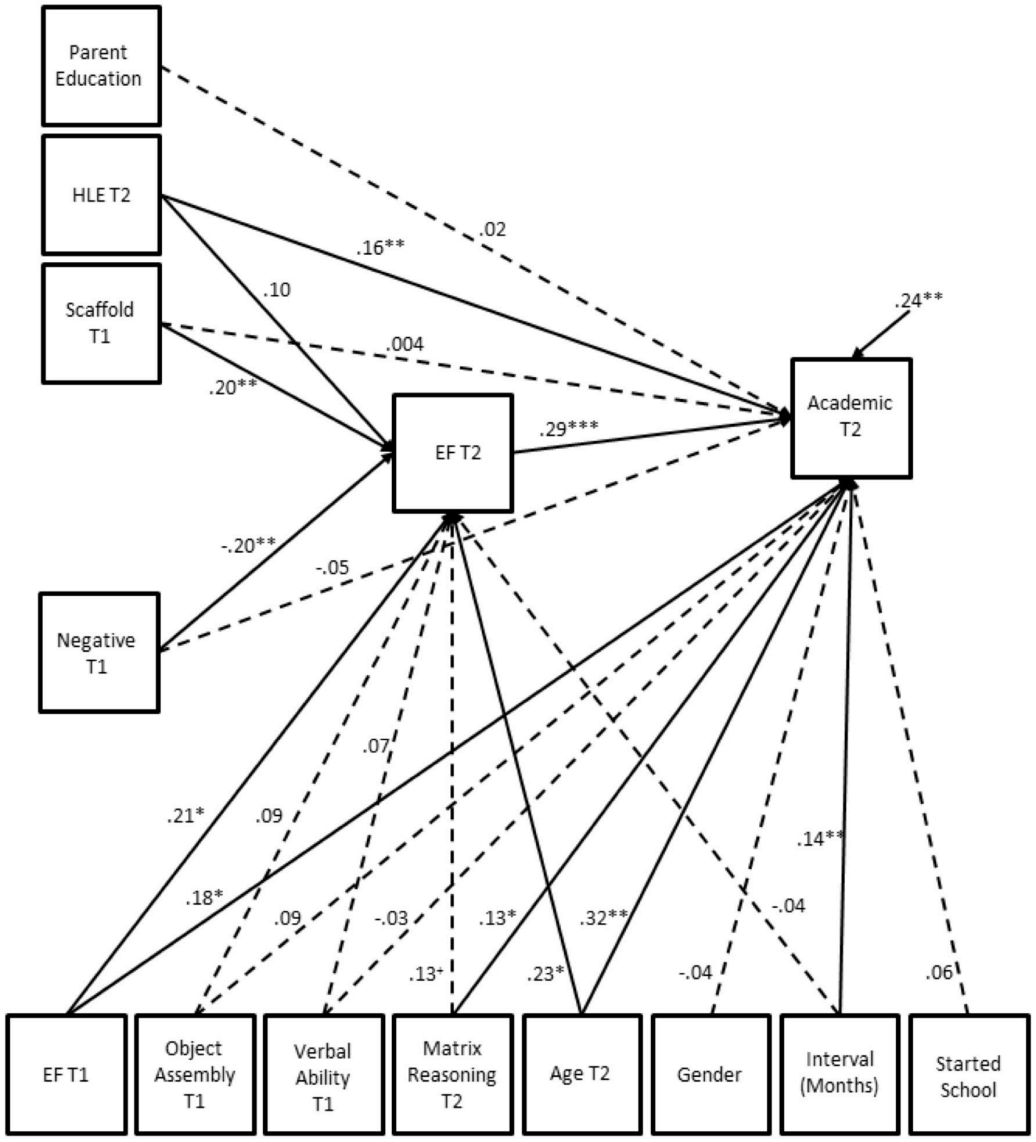
**Standardized robust maximum likelihood estimates for longitudinal mediation model**. ^***^p < 0.001. ^**^p < 0.0l. ^*^p < 0.05. ^+^*p* < 0.10. Dashed lines are non-significant paths. Solid lines represent significant paths. HLE, Home Learning Environment; Scaffold, Parental Use of Contingency Rule; Negative, Negative Parent-Child Interactions; EF, Executive Function. Academic, Composite Academic Ability Score; Interval, Time between T1 and T2 in months; Started School, Whether or not the child has been in formal education. T1, Time 1. T2, Time 2.

We specified two longitudinal models to examine the direct and indirect effects (via EF and performance on the Matrix Reasoning task) of parental behavior on children's early academic ability. In the first model, academic ability was regressed onto measures of EF (at Time 1 and Time 2) and each measure of parental behavior. Note that, by regressing academic ability onto EF at Time 1 and Time 2 and regressing EF at Time 2 onto EF at Time 1, we were able to disentangle whether early EF made a unique contribution to later academic ability controlling for concurrent EF (at Time 2). In addition we controlled statistically for the influence of verbal ability and general cognitive ability (as measured by performance on the Object Assembly and Matrix Reasoning tasks), parental education (as measured by a dummy variable with 0 indicating no degree and 1 indicating achievement of an undergraduate degree), gender (using a dummy code of 0 for girls and 1 for boys), whether the child had started formal schooling at Time 2 (using a dummy code with 0 indicating no and 1 indicating yes), the interval between Time 1 and Time 2 (in months) and child age by regressing both academic ability and EF at Time 2 on these variables. Each of the predictor variables in our model were free to co-vary. This first model provided an acceptable fit to the data: $\chi_{\left(3\right)}^{2}$ = 0.89, *p* = 0.83, CFI = 1.00, TLI = 1.09, RMSEA = 0. Standardized path estimates for this model are shown in Figure 2. Unstandardized estimates and 95% confidence intervals for all model parameters are presented in Table 3. The overall model accounted for 76% of the variance in children's academic ability. EF at Time 1 and Time 2 were moderately and significantly related to academic ability uniquely accounting for 2 and 4% of the variance respectively.

**Table 3:** **Unstandardized and standardized robust maximum likelihood parameter estimates for longitudinal mediation model 1**.

**Model parameter**	**Unstandardized estimate (SE)**	**Standardized estimate**	**95% CI standardized**
**ACADEMIC ABILITY ON**			
Executive function T2	0.383 (0.086)	0.29^**^	[0.17, 0.40]
Executive function T1	0.215(0.088)	0.18^*^	[0.06, 0.30]
Verbal ability T1	−0.062 (0.117)	−0.03	[−0.14, 0.07]
Matrix reasoning T2	0.313 (0.113)	0.13^*^	[0.04, 0.23]
Object assembly T1	0.107 (0.066)	0.09	[−0.001, 0.19]
Age T2	5.393 (1.428)	0.32^**^	[0.18, 0.45]
Commenced formal Schooling	1.285 (1.310)	0.06	[−0.04, 0.17]
Gender	−0.681 (1.114)	−0.04	[−0.13, 0.07]
Parental education	0.568 (1.420)	0.02	[−0.07, 0.12]
Parental use of contingency rule	0.167 (2.349)	0.01	[−0.09, 0.08]
Negative parent-child interaction	−0.059 (0.050)	−0.05	[−0.12, 0.02]
Home learning environment	0.164 (0.059)	0.16^**^	[0.07, 0.26]
Testing interval (months)	0.794 (0.280)	0.14^**^	[0.06, 0.22]
**EXECUTIVE FUNCTION T2 ON**			
Executive function T1	0.190 (0.079)	0.21^**^	[0.07, 0.35]
Verbal ability T1	0.089 (0.141)	0.07	[−0.10, 0.23]
Matrix reasoning T2	0.189 (0.114)	0.13^+^	[0.003, 0.26]
Object assembly T1	0.076 (0.069)	0.10	[−0.04, 0.22]
Age T2	2.943 (1.457)	0.23^*^	[0.05, 0.41]
Parental use of contingency rule	7.005 (2.466)	0.20^**^	[0.09, 0.31]
Negative parent-child interaction	−0.174 (0.068)	−0.20^**^	[−0.31, −0.08]
Home learning environment	0.078 (0.059)	0.10	[−0.03, 0.23]
Testing interval (months)	−0.186 (0.349)	−0.04	[−0.18, 0.09]
**EXECUTIVE FUNCTION T1 WITH**			
Verbal ability T1	14.511 (3.621)	0.37^**^	[0.24, 0.50]
Matrix reasoning T2	11.670 (3.451)	0.31^**^	[0.18, 0.45]
Object assembly T1	26.987 (5.524)	0.43^**^	[0.31, 0.55]
Age T2	2.629 (0.361)	0.62^**^	[0.52, 0.72]
Commenced formal schooling	1.645 (0.346)	0.47^**^	[0.33, 0.61]
Gender	−0.350 (0.354)	−0.09	[−0.24, 0.06]
Parental education	0.492 (0.335)	0.16	[−0.01, 0.34]
Parental use of contingency rule	0.280 (0.161)	0.18^+^	[0.01, 0.34]
Negative parent-child interaction	−4.111 (5.299)	−0.07	[−0.22, 0.08]
Home learning environment	1.950 (6.986)	0.03	[−0.14, 0.19]
Testing interval (months)	1.300 (1.300)	0.10	[−0.07, 0.27]
**VERBAL ABILITY T1 WITH**			
Matrix reasoning T2	9.415 (2.368)	0.38^**^	[0.26, 0.51]
Object assembly T1	16.201 (4.094)	0.39^**^	[0.25, 0.52]
Age T2	1.451 (0.278)	0.52^**^	[0.39, 0.64]
Commenced formal schooling	0.560 (0.242)	0.24^*^	[0.08, 0.40]
Gender	−0.398 (0.233)	−0.16^+^	[−0.30, −0.01]
Parental education	0.299 (0.189)	0.15	[−0.01, 0.30]
Parental use of contingency rule	0.132 (0.109)	0.13	[−0.04, 0.31]
Negative parent-child interaction	−9.404 (3.694)	−0.23^*^	[−0.39, −0.08]
Home learning environment	2.094 (4.810)	0.05	[−0.13, 0.19]
Testing interval (months)	−0.569 (0.843)	−0.07	[−0.23, 0.10]
**MATRIX REASONING T2 WITH**			
Object assembly T1	17.048 (4.154)	0.43^**^	[0.29, 0.57]
Age T2	1.286 (0.263)	0.48^**^	[0.36, 0.60]
Commenced formal schooling	0.356 (0.212)	0.16^+^	[0.01, 0.32]
Gender	0.118 (0.235)	0.05	[−0.11, 0.21]
Parental education	0.365 (0.154)	0.19^**^	[−0.10, 0.21]
Parental use of contingency rule	−0.048 (0.089)	−0.06	[−0.21, 0.09]
Negative parent-child interaction	−4.301 (4.176)	−0.11	[−0.30, 0.08]
Home learning environment	7.563 (4.164)	0.17^+^	[0.03, 0.32]
Testing interval (months)	1.063 (0.866)	0.13	[−0.04, 0.31]
**OBJECT ASSEMBLY T1 WITH**			
Age T2	2.400 (0.413)	0.53^**^	[0.42, 0.64]
Commenced formal schooling	1.356 (0.340)	0.36^**^	[0.23, 0.50]
Gender	0.399 (0.378)	0.10	[−0.05, 0.25]
Parental education	0.385 (0.260)	0.12	[−0.01, 0.25]
Parental use of contingency rule	0.011 (0.177)	0.01	[−0.17, 0.18]
Negative parent-child interaction	−16.383 (5.736)	−0.25^**^	[−0.39, −0.12]
Home learning environment	−2.342 (7.760)	−0.03	[−0.20, 0.14]
Testing interval (months)	−0.007 (1.309)	−0.01	[−0.16, 0.16]
**AGE T2 WITH**			
Commenced formal schooling	0.133 (0.025)	0.53^**^	[0.39, 0.67]
Gender	0.014 (0.601)	0.05	[−0.11, 0.21]
Parental education	−0.001 (0.020)	−0.01	[−0.16, 0.15]
Parental use of contingency rule	0.008 (0.011)	0.06	[−0.10, 0.23]
Negative parent-child interaction	−0.258 (0.442)	−0.05	[−0.23, 0.11]
Home learning environment	0.014 (0.500)	0.01	[−0.16, 0.17]
Testing interval (months)	0.213 (0.088)	0.24^*^	[0.08, 0.39]
**COMMENCED FORMAL SCHOOLING WITH**			
Gender	−0.003 (0.022)	−0.01	[−0.17, 0.15]
Parental education	0.008 (0.675)	0.05	[−0.13, 0.22]
Parental use of contingency rule	−0.003 (0.009)	−0.04	[−0.20, 0.13]
Negative parent-child interaction	0.383 (0.327)	0.11	[−0.04, 0.25]
Home learning environment	−0.086 (0.427)	−0.02	[−0.19, 0.15]
Testing interval (months)	0.101 (0.077)	0.14	[−0.03, 0.30]
**GENDER WITH**			
Parental education	0.011 (0.018)	0.06	[−0.10, 0.21]
Parental use of contingency rule	−0.007 (0.010)	−0.07	[−0.23, 0.09]
Negative parent-child interaction	0.729 (0.364)	0.19^*^	[0.04, 0.33]
Home learning environment	–1.110 (0.437)	−0.24^*^	[−0.40, −0.09]
Testing interval (months)	0.087 (0.080)	0.11	
**PARENTAL EDUCATION WITH**			
Parental use of contingency rule	0.016 (0.007)	0.21^**^	[0.07, 0.34]
Negative parent-child interaction	0.129 (0.215)	0.04	[−0.07, 0.15]
Home learning environment	0.162 (0.389)	0.05	[−0.13, 0.22]
Testing interval (months)	−0.035 (0.069)	−0.06	[−0.05, 0.27]
**PARENTAL SCAFFOLDING WITH**			
Negative parent-child interaction	−0.185 (0.187)	−0.12	[−0.32, 0.07]
Home learning environment	0.123 (0.172)	0.07	[−0.09, 0.22]
Testing interval (months)	0.041 (0.034)	0.13	
**NEGATIVE PARENT-CHILD INTERACTION WITH**			
Home learning environment	11.715 (7.643)	0.16^+^	[0.01, 0.32]
Testing interval (months)	−0.561 (1.396)	−0.04	[−0.04, 0.29]
**HOME LEARNING ENVIRONMENT WITH**			
Testing interval (months)	−2.711 (1.427)	−0.18^+^	[−0.33, −0.03]

** *p < 0.01*.

* *p < 0.05*.

+ *p < 0.10. On, Regressed onto; With, Correlated with*.

Parental scaffolding and negative parent-child interaction uniquely accounted for 5 and 4% of the variance in Time 2 EF but only 0.1 and 0.2% of the variance in academic ability. Statistical tests of indirect effects revealed that EF at Time 2 mediated the relations between negative parent-child interactions and academic ability, *B* = −0.07, *SE* = 0.03, *Z* = −2.25, *p* = 0.024, and between parental scaffolding and academic ability, *B* = 2.68, *SE* = 1.13, *Z* = 2.38, *p* = 0.017. These findings were confirmed by the non-significant direct path between negative parent-child interaction and academic ability, *B* = −0.06, *SE* = 0.05, *Z* = −1.18, *p* = 0.24, β = −0.05, and between parental scaffolding and academic ability, *B* = 0.17, *SE* = 2.34, *Z* = 0.07, *p* = 0.94, β = 0.01. EF did not mediate the link between the HLE and academic ability, *B* = 0.03, *SE* = 0.02, *Z* = 1.36, *p* = 0.17. Instead there was significant direct relation between the HLE and academic ability, *B* = 0.16, *SE* = 0.06, *Z* = 2.76, *p* = 0.005, β = 16. HLE uniquely accounted for 1% of the variance in academic ability.

To examine the specificity of EF as a mediator of the effects of negative parent-child interaction and parental scaffolding on academic ability, we tested a second longitudinal model in which general cognitive ability (as measured by the Matrix Reasoning task) was entered as a mediator between negative parent-child interaction, parental scaffolding and academic ability instead of EF. As before, we controlled statistically for the influence of general cognitive ability at Time 1, EF at Time 1 and Time 2, parental education, formal schooling, gender, and age by regressing the dependent variable and mediator on each of these covariates. This second model provided an acceptable fit to the data on three out of four indices: $\chi_{\left(7\right)}^{2}$ = 11.64, *p* = 0.11, CFI = 0.98, TLI = 0.90, RMSEA = 0.07. Examination of the tests of indirect effects revealed that general cognitive ability at Time 2 (as measured by the Matrix Reasoning task) did not mediate the relation between negative parent-child interaction and academic achievement, *B* = −0.01, *SE* = 0.02, *Z* = −0.63, *p* = 0.53, or the link between parental scaffolding and academic achievement, *B* = −0.83, *SE* = 0.73, *Z* = −1.13, *p* = 0.26. To summarize, our models revealed three key sets of findings. Firstly, the three different aspects of parental behavior were not significantly correlated with each other. Secondly, individual differences in EF (measured at both Time 1 and Time 2) showed unique relations with children's academic ability. Thirdly, EF mediated the links between negative parent-child interaction and academic ability on the one hand and between parental scaffolding and academic ability on the other hand. Variation in the HLE, however, was directly related to early academic ability.

## Discussion

This longitudinal study of 117 parent-child dyads makes at least three contributions to the existing literature. First, supporting a differentiated model of parenting (e.g., Carr and Pike, 2012), different aspects of parental behavior were unrelated to each other and showed unique contributions to children's early academic ability. Second, our analyses showed that children's EF mediated the relations between parental scaffolding and negative parent-child interaction and children's early academic ability. Third, our results revealed that EF and not general cognitive ability (as measured by the Matrix Reasoning task) mediated the relations between these two aspects of parental behavior and children's academic ability.

With some notable exceptions (e.g., Hughes and Ensor, 2009; Bernier et al., 2010), existing studies of parental influences on children's academic ability and on children's EF have typically either focused on a single aspect of parenting or adopted a global approach by aggregating several domains of parental behavior into a single measure. While these studies have been valuable in highlighting the influence of parental behaviors on children's cognitive and academic abilities, progress in understanding the mechanisms underpinning these associations has been limited by the scarcity of studies seeking to disentangle the relations between different aspects of parental behavior and child outcomes.

In response to this challenge, we followed calls for fine-grained analyses (e.g., Davidov and Grusec, 2006; Carr and Pike, 2012) by distinguishing three aspects of parental behavior (i.e., parental scaffolding, negative parent-child interaction and provision of opportunities for learning) that have been studied in relation to children's academic ability and EF. Our results showed that these three dimensions of parental behavior were unrelated, but each dimension exhibited weak associations with individual differences in children's academic ability (even when age and general cognitive ability were taken into account). It is possible that our measure of the HLE was unrelated to our measures of parental scaffolding and negative parent-child interaction because these constructs were measured in very different ways (i.e., observation vs. questionnaire). That said, our two observational measures were also unrelated to each other. It would therefore be valuable in future studies to include multiple indicators of each aspect of parental behavior to understand the structure of this differentiated model of parenting more fully.

The main goal of our study was to elucidate the mechanisms by which parental behaviors are related to children's early academic abilities. In doing so, we outlined three theoretical models linking parental behavior, children's EF and academic ability. The first of these models, the Domain General Model, suggests that a range of parental behaviors will exhibit direct associations with a range of cognitive outcomes. That is, parents who provide high levels of cognitive support, frequent opportunities for engagement in informal learning activities and low levels of negative parent-child interaction, will have children who perform better across the board. The second of these models, the Domain Specific Model, proposes that specific parental behaviors will be directly linked with specific cognitive outcomes. For example, frequent engagement in informal literacy and numeracy activities will be associated with better academic performance and children exposed to parent-child interactions characterized by contingency and low levels of negativity will exhibit superior EF. The third model, the Mediation Model, suggests that parental behaviors indirectly influence children's academic ability via more specific cognitive mechanisms (e.g., EF or general cognitive ability). Our findings show that these different models are not mutually exclusive. The relations between two aspects of parental behavior (i.e., parental scaffolding and negative parent-child interaction) and children's academic ability were mediated by children's EF. In contrast, informal opportunities for learning (as measured by the HLE questionnaire) exhibited direct effects on children's academic ability. Importantly, for the first time, our findings showed that EF and not general cognitive ability played a specific role in the relation between parental scaffolding, negative parent-child interaction and children's academic ability.

Before discussing these findings, a number of limitations in our study deserve note. First, our longitudinal study involved just two time points. Numerous theorists have argued that two-wave or “half longitudinal” designs (in which the mediator is measured at the same time point as either the predictor or outcome variable) are a cost-effective way to examine mediation and are preferable to more widely-used cross-sectional designs (Cole and Maxwell, 2003; Little et al., 2007; Newsom, 2015; Preacher, 2015). Although the existing findings on the relations between parental behavior, EF and academic ability reported earlier involved multiple time points, the presumed mediator was either measured alongside the predictor (e.g., NICHD Early Child Care Research Network, 2003) or the outcome (Friedman et al., 2014). Future studies involving three (or more) time points in which the parental behaviors, EF and academic outcomes were measured at different time points would permit the underlying assumptions of stationarity and equilibrium to be tested formally (Cole and Maxwell, 2003). Second, our longitudinal study involved assessment of parental behavior at just one time point (i.e., parent-child interactions were studied at Time 1 only and parental reports of the HLE were gathered at Time 2 only) and so cross-lagged analyses to determine the direction of the association between parental behavior, EF and academic outcomes was not possible (Menard, 2002). Third, academic ability was measured at just one time point. Ideally, auto-regressive models require that the dependent variable should be measured on at least two occasions so that stability in the dependent variable can be accounted for (Hertzog and Nesselroade, 2003). However, we took steps to reduce potential confounds by including a range of covariates in our models and controlled for individual differences in earlier verbal ability, general cognitive ability and EF (as well as parental education, child age, and formal schooling) in each of our models.

Notwithstanding these limitations, our results complement those based on the NICHD study demonstrating that individual differences in EF mediate the relation between parental behavior and children's later academic achievement (NICHD Early Child Care Research Network, 2003; Friedman et al., 2014) and extend that work by disentangling the relative influence of different dimensions of parental behavior and demonstrating the specificity of EF as a mediator. Moreover, our findings are also consistent with a growing body of research showing that children's EF mediates the relations between harsh or insensitive parental behavior, maternal depressive symptoms and children's externalizing problems (Sulik et al., 2015; Roman et al., 2016). While not focused on academic outcomes these studies provide a template for future longitudinal research on parental behavior, EF and children's academic ability by: (1) spanning more than two time points so that formal tests of mediation can be carried out; (2) incorporating measures of each construct at every time point to unpack the temporal dynamics of these associations (Sulik et al., 2015); and (3) testing alternative mediators to determine the specificity of EF as a mediator (Roman et al., 2016).

Causal claims about the purported developmental relations between parental behavior, EF and children's early academic ability will be bolstered by intervention and genetically sensitive studies. There is now considerable evidence that parental behaviors can be modified through a range of interventions (e.g., Kaminski et al., 2008; Belsky and de Haan, 2011). Moreover, studies of the impact of school-based interventions to improve children's academic outcomes suggest that the effects of these programs are mediated by EF (Raver et al., 2011). Whether or not parent-focused interventions exert effects on child outcomes via EF remains to be seen but such evidence would provide support for any causal claims about the relations between parental behavior, children's EF and early academic ability. When parents and children are biologically related, longitudinal studies of parental effects on children's cognition are potentially confounded by genetic effects (Dale et al., 2015). Indeed a number of twin studies suggest that individual differences in EF show substantial heritability in middle childhood, adolescence, and adulthood (e.g., Polderman et al., 2007; Friedman et al., 2008). Moreover, a large-scale study using Genome-Wide Complex Trait Analysis (GCTA) has shown that genetic factors accounted for the relations between family socio-economic status (SES) and children's IQ at ages 7 and 12 (Trzaskowski et al., 2014) and between SES and children's educational achievement (Krapohl and Plomin, 2016). Genetically sensitive research designs (e.g., adoption studies) will help to disentangle genetic and environmental effects on children's EF and early academic ability. In addition to this work, investigations of potential moderating variables will also elucidate the mechanisms by which parental behaviors shape early academic abilities. For example, researchers have identified specific DNA polymorphisms related to the signaling of dopamine that moderate children's susceptibility to parental influences on a variety of cognitive and behavioral outcomes (Bakermans-Kranenburg and Van Ijzendoorn, 2011). It is conceivable that genetic factors might act to attenuate or strengthen the developmental relations between parental behaviors, children's EF and academic abilities.

## Summary and conclusion

We have shown that individual differences in children's EF (but not general cognitive ability) mediate the relations between each of two aspects of parental behavior (that is, “parental scaffolding” or the proclivity to modify instructions and support in response to children's behavior and “negative parent-child interaction” or the extent to which parents are critical, controlling and display negative affect on the other) and children's early academic ability. That is, parental scaffolding and negative parent-child interaction appear to influence children's academic abilities by helping or hindering children's emerging EF. In contrast, parental provision of opportunities for learning in the home environment is directly related to children's academic abilities. Future studies on the relations between parental behaviors, children's EF and early academic abilities will benefit from adopting multi-wave longitudinal and training designs as well as a find-grained approach to studying the relative salience of different aspects of parental behavior.

## Ethics statement

University of Cambridge Psychology Research Ethics Committee. Participants' parents/caregivers were issued with leaflets explaining the nature of the study in detail. We provided parents/caregivers with our contact information so they did not have to make a choice to participate immediately. Parents had the opportunity to ask questions before consenting to participate. Upon agreeing to participate, parents provided written consent before taking part in a testing session. Children were closely monitored for signs of distress and discomfort during the testing procedures. Breaks were provided and children were issued with small rewards (e.g., stickers) and praise regardless of their task performance.

## Author contributions

RD and CH shared responsibility for the conception, design, interpretation, and write up of this study. In addition RD undertook all data collection and data analysis. GB shared responsibility with RD for coding parent-child interactions.

## Funding

This study was funded by the UK Economic and Social Research Council (ES/JO21180/1) and the Isaac Newton Trust, Cambridge.

### Conflict of interest statement

The authors declare that the research was conducted in the absence of any commercial or financial relationships that could be construed as a potential conflict of interest.

## References

[B1] Acock A. C. (2005). Working with missing values. J. Marriage Fam. 67, 1012–1028. 10.1111/j.1741-3737.2005.00191.x

[B2] Asparouhov T. Muthén B. (2010). Weighted Least Squares Estimation with Missing Data. MPlus Technical Appendix.

[B3] Bakermans-Kranenburg M. J. Van Ijzendoorn M. H. (2011). Differential susceptibility to rearing environment depending on dopamine-related genes: new evidence and a meta-analysis. Dev. Psychopathol. 23, 39–52. 10.1017/S0954579410000635 21262038

[B4] Belsky J. de Haan M. (2011). Parenting and children's brain development: the end of the beginning. J. Child Psychol. Psychiatry 52, 409–428. 10.1111/j.1469-7610.2010.02281.x 20626527

[B5] Bernier A. Carlson S. M. Whipple N. (2010). From external regulation to self-regulation: early parenting precursors of executive functioning. Child Dev. 81, 326–339. 10.1111/j.1467-8624.2009.01397.x 20331670

[B6] Blair C. Granger D. A. Willoughby M. Mills-Koonce R. Cox M. Greenberg M. T.. (2011). Salivary cortisol mediates effects of poverty and parenting on executive functions in early childhood. Child Dev. 82, 1970–1984. 10.1111/j.1467-8624.2011.01643.x 22026915 PMC3218241

[B7] Blair C. Raver C. C. (2015). School readiness and self-regulation: a developmental psychobiological approach. Annu. Rev. Psychol. 66, 711–731. 10.1146/annurev-psych-010814-015221 25148852 PMC4682347

[B8] Blair C. Razza R. P. (2007). Relating effortful control, executive function, and false belief understanding to emerging math and literacy ability in kindergarten. Child Dev. 78, 647–663. 10.1111/j.1467-8624.2007.01019.x 17381795

[B9] Bradley R. H. Caldwell B. M. Corwyn R. F. (2003). The child care HOME inventories: assessing the quality of family childcare homes. Early Child. Res. Q. 18, 294–309. 10.1016/S0885-2006(03)00041-3

[B10] Bradley R. H. Caldwell B. M. Elardo R. (1979). Home environment and cognitive development in the first 2 years: a cross-lagged panel analysis. Dev. Psychol. 15, 246–250. 10.1037/0012-1649.15.3.246

[B11] Bradley R. H. Caldwell B. M. Rock S. L. (1988). Home environment and school performance: a ten-year follow-up and examination of three models of environmental action. Child Dev. 59, 852–867. 10.2307/1130253 3168624

[B12] Brown T. A. (2015). Confirmatory Factor Analysis for Applied Research, 2nd Edn. London: Guilford Press.

[B13] Carr A. Pike A. (2012). Maternal scaffolding behavior: links with parenting style and maternal education. Dev. Psychol. 48, 543–551. 10.1037/a0025888 22004338

[B14] Clark C. A. Sheffield T. D. Wiebe S. A. Espy K. A. (2013). Longitudinal associations between executive control and developing mathematical competence in preschool boys and girls. Child Dev. 84, 662–677. 10.1111/j.1467-8624.2012.01854.x 23006040 PMC3530644

[B15] Cohen J. (1988). Statistical Power Analysis for the Behavioral Sciences, 2nd Edn. Hillsdale, NJ: Lawrence Erlbaum.

[B16] Cole D. A. Maxwell S. E. (2003). Testing mediational models with longitudinal data: questions and tips in the use of structural equation modelling. J. Abnorm. Psychol. 112, 558–577. 10.1037/0021-843X.112.4.558 14674869

[B17] Conner D. B. Knight D. K. Cross D. R. (1997). Mothers' and fathers' scaffolding of their 2-year-olds during problem-solving and literacy interactions. Br. J. Dev. Psychol. 15, 323–338. 10.1111/j.2044-835X.1997.tb00524.x

[B18] Cragg L. Nation K. (2007). Self-ordered pointing as a test of working memory in typically developing children. Memory 15, 526–535. 10.1080/09658210701390750 17613795

[B19] Culp A. M. Tait L. H. Culp R. E. Starost H. J. (2000). Maternal parenting characteristics and school involvement: predictors of Kindergarten cognitive competence among Head Start Children. J. Res. Childhood Educ. 15, 5–17. 10.1080/02568540009594772

[B20] Dale P. S. Grazia-Tosto M. Hayiou-Thomas M. Plomin R. (2015). Why does parental language input style predict child language development? A twin study of gene-environment correlation. J. Commun. Disord. 57, 106–117. 10.1016/j.jcomdis.2015.07.004 26277213 PMC4610950

[B21] Davidov M. Grusec J. E. (2006). Untangling the links of parental responsiveness to distress and warmth to child outcomes. Child Dev. 77, 44–58. 10.1111/j.1467-8624.2006.00855.x 16460524

[B22] Deater-Deckard K. Pylas M. Petrill S. A. (1997). The Parent-Child Interaction System (PARCHISY). London: Institute of Psychiatry.

[B23] Diamond A. (2013). Executive functions. Annu. Rev. Psychol. 64, 135–168. 10.1146/annurev-psych-113011-143750 23020641 PMC4084861

[B24] Duncan G. J. Dowsett C. J. Claessens A. Magnuson K. Huston A. C. Klebanov P.. (2007). School readiness and later achievement. Dev. Psychol. 43, 1428–1446. 10.1037/0012-1649.43.6.1428 18020822

[B25] Enders C. K. (2001). A primer on maximum likelihood algorithms available for use with missing data. Struct. Equ. Modeling, 8, 128–141. 10.1207/S15328007SEM0801_7

[B26] Espy K. A. McDiarmid M. M. Cwik M. F. Stalets M. M. Hamby A. Senn T. E. (2004). The contribution of executive functions to emergent mathematic skills in preschool children. Dev. Neuropsychol. 26, 465–486. 10.1207/s15326942dn2601_6 15276905

[B27] Fitzpatrick C. McKinnon R. D. Blair C. B. Willoughby M. T. (2014). Do preschool executive function skills explain the school readiness gap between advantaged and disadvantaged children? Learn. Instruct. 30, 25–31. 10.1016/j.learninstruc.2013.11.003

[B28] Friedman N. P. Miyake A. Young S. E. Defries J. C. Corley R. P. Hewitt J. K. (2008). Individual differences in executive functions are almost entirely genetic in origin. J. Exp. Psychol. Gen. 137, 201–225. 10.1037/0096-3445.137.2.201 18473654 PMC2762790

[B29] Friedman S. L. Scholnick E. K. Bender R. H. Vandergrift N. Spieker S. Hirsh Pasek K.. (2014). Planning in middle childhood: early predictors and later outcomes. Child Dev. 85, 1446–1460. 10.1111/cdev.12221 24476334

[B30] Fuhs M. W. Nesbitt K. T. Farran D. C. Dong N. (2014). Longitudinal associations between executive functioning and academic skills across content areas. Dev. Psychol. 50, 1698–1709. 10.1037/a0036633 24749550

[B31] Gerstadt C. L. Hong Y. J. Diamond A. (1994). The relationship between cognition and action: performance of children 3 1/2−7 years old on a Stroop-like day-night test. Cognition 53, 129–153. 10.1016/0010-0277(94)90068-X 7805351

[B32] Hammond S. I. Müller U. Carpendale J. I. M. Bibok M. B. Lieberman-Finestone D. P. (2012). The effects of parental scaffolding on preschoolers' executive function. Dev. Psychol. 48, 271–281. 10.1037/a0025519 21928877

[B33] Harold G. T. Aitken J. J. Shelton K. H. (2007). Inter-parental conflict and children's academic attainment: a longitudinal analysis. J. Child Psychol. Psychiatry 48, 1223–1232. 10.1111/j.1469-7610.2007.01793.x 18093028

[B34] Hertzog C. Nesselroade J. R. (2003). Assessing psychological change in adulthood. Psychol. Aging 18, 639–657. 10.1037/0882-7974.18.4.639 14692854

[B35] Hindman A. H. Morrison F. J. (2012). Differential contributions of three parenting dimensions to preschool literacy and social skills in a middle-income sample. Merrill Palmer Q. 58, 191–223. 10.1353/mpq.2012.0012

[B36] Hughes C. Ensor R. (2005). Executive function and theory of mind in 2 year olds: a family affair? Dev. Neuropsychol. 28, 645–668. 10.1207/s15326942dn2802_5 16144431

[B37] Hughes C. Roman G. Hart M. J. Ensor R. (2013). Does maternal depression predict young children's executive function? A 4-year longitudinal study. J. Child Psychol. Psychiatry 54, 169–177. 10.1111/jcpp.12014 23171379

[B38] Hughes C. H. Ensor R. A. (2009). How do families help or hinder the emergence of early executive function? New Dir. Child Adolesc. Dev. 123, 35–50. 10.1002/cd.234 19306273

[B39] Jimerson S. Egeland B. Sroufe A. Carlson B. (2000). A prospective longitudinal study of high school dropouts: examining multiple predictors across development. J. Sch. Psychol. 38, 525–549. 10.1016/S0022-4405(00)00051-0

[B40] Kaminski J. W. Valle L. A. Filene J. H. Boyle C. L. (2008). A meta-analytic review of components associated with parent training program effectiveness. J. Abnorm. Child Psychol. 36, 567–589. 10.1007/s10802-007-9201-9 18205039

[B41] Kluczniok K. Lehrl S. Kuger S. Rossbach H. (2013). Quality of the home learning environment during preschool age: domains and contextual conditions. Eur. Early Childhood Educ. Res. J. 21, 420–438. 10.1080/1350293X.2013.814356

[B42] Krapohl E. Plomin R. (2016). Genetic link between family socioeconomic status and children's educational achievement estimated from genome-wide SNPs. Mol. Psychiatry 21, 437–443. 10.1038/mp.2015.2 25754083 PMC4486001

[B43] Lagattuta K. H. Sayfan L. Monsour M. (2011). A new measure for assessing executive function across a wide age range: children and adults find happy-sad more difficult than day-night. Dev. Sci. 14, 481–489. 10.1111/j.1467-7687.2010.00994.x 21477188

[B44] Lamb Parker F. Boak A. Y. Griffin K. W. Ripple C. Peay L. (1999). Parent-child relationship, home learning environment, and school readiness. School Psych. Rev. 28, 413–425.

[B45] La Paro K. M. Pianta R. C. (2000). Predicting children's competence in the early school years: a meta-analytic review. Rev. Educ. Res. 70, 443–484. 10.3102/00346543070004443

[B46] Leerkes E. M. Blankson A. N. O'Brien M. Calkins S. D. Marcovitch S. (2011). The relation of maternal emotional and cognitive support during problem solving to pre-academic skills in preschoolers. Infant Child Dev. 20, 353–370. 10.1002/icd.728 22121336 PMC3222582

[B47] Lehrl S. Kluczniok K. Rossbach H. (2016). Long-term associations of preschool education: the predictive role of preschool quality for the development of mathematical skills through elementary school. Early Child. Res. Q, 36, 475–488. 10.1016/j.ecresq.2016.01.013

[B48] Little T. D. Preacher K. J. Selig J. P. Card N. A. (2007). New developments in latent variable panel analysis of longitudinal data. Int. J. Behav. Dev. 31, 357–365. 10.1177/0165025407077757

[B49] Mattanah J. F. Pratt M. W. Cowan P. A. Cowan C. P. (2005). Authoritative parenting, parental scaffolding of long-division mathematics, and children's academic competence in fourth grade. Appl. Dev. Psychol. 26, 85–106. 10.1016/j.appdev.2004.10.007

[B50] Meins E. (1997). Security of attachment and maternal tutoring strategies: interaction within the zone of proximal development. Br. J. Dev. Psychol. 15, 129–144. 10.1111/j.2044-835X.1997.tb00730.x

[B51] Melhuish E. C. Sylva K. Sammons P. Siraj-Blatchford I. Taggart B. Phan M. B.. (2008). Preschool influences on mathematics achievement. Science 321, 1161–1162. 10.1126/science.1158808 18755959

[B52] Menard S. (2002). Longitudinal Research, 2nd Edn. London: Sage.

[B53] Miyake A. Friedman N. P. (2012). The nature and organization of individual differences in executive functions: four general conclusions. Curr. Dir. Psychol. Sci. 21, 8–14. 10.1177/0963721411429458 22773897 PMC3388901

[B54] Müller U. Baker L. Yeung E. (2013). A developmental systems approach to executive function. Adv. Child Dev. Behav. 45, 39–66. 10.1016/B978-0-12-397946-9.00003-8 23865112

[B55] Muthén L. K. Muthén B. (2012). MPlus: Statistical Analysis with Latent Variables User Guide, 7th Edn. Los Angeles, CA: Muthen and Muthen.

[B56] Neitzel C. Stright A. D. (2003). Mothers' scaffolding of children's problem solving: establishing a foundation of academic self-regulatory competence. J. Fam. Psychol. 17, 147–159. 10.1037/0893-3200.17.1.147 12666470

[B57] Nesbitt K. T. Farran D. C. Fuhs M. W. (2015). Executive function skills and academic achievement gains in prekindergarten: contributions of learning-related behaviors. Dev. Psychol. 51, 865–878. 10.1037/dev0000021 26010383

[B58] Newsom J. T. (2015). Longitudinal Structural Equation Modelling. London: Routledge.

[B59] NICHD Early Child Care Research Network (2003). Do children's attention processes mediate the link between family predictors and school readiness? Dev. Psychol. 39, 581–593. 10.1037/0012-1649.39.3.581 12760525

[B60] NICHD Early Child Care Research Network (2008). Mothers' and fathers' support for child autonomy and early school achievement. Dev. Psychol. 44, 895–907. 10.1037/0012-1649.44.4.895 18605822

[B61] Pettit G. S. Bates J. E. Dodge K. A. (1997). Supportive parenting, ecological context and children's adjustment: a seven-year longitudinal study. Child Dev. 68, 908–923. 10.2307/1132041 29106716

[B62] Polderman T. J. C. Posthuma D. De Sonneville L. M. J. Stins J. F. Verhulst F. C. Boomsma D. I. (2007). Genetic analysis of the stability of executive functioning during childhood. Biol. Psychol. 76, 11–20. 10.1016/j.biopsycho.2007.05.002 17597285

[B63] Pratt M. W. Green D. MacVicar J. Bountrogianni M. (1992). The mathematical parent: parental scaffolding, parenting style and learning outcomes in long-division mathematics homework. J. Appl. Dev. Psychol. 13, 17–34. 10.1016/0193-3973(92)90003-Z

[B64] Pratt M. W. Kerig P. Cowan P. A. Cowan C. P. (1988). Mothers and fathers teaching 3-year-olds: authoritative parenting and adult scaffolding of young children's learning. Dev. Psychol. 24, 832–839. 10.1037/0012-1649.24.6.832

[B65] Preacher K. J. (2015). Advances in mediation analysis: a survey and synthesis of new developments. Annu. Rev. Psychol. 66, 825–852. 10.1146/annurev-psych-010814-015258 25148853

[B66] Raver C. C. Jones S. M. Li-Grining C. Zhai F. Bub K. Pressler E. (2011). CSRP's impact on low-income preschoolers' preacademic skills: self-regulation as a mediating mechanism. Child Dev. 82, 362–378. 10.1111/j.1467-8624.2010.01561.x 21291447 PMC3682645

[B67] Roman G. D. Ensor R. Hughes C. (2016). Does executive function mediate the path from mothers' depressive symptoms to young children's problem behaviors? J. Exp. Child Psychol. 142, 158–170. 10.1016/j.jecp.2015.09.022 26550956

[B68] Roth B. Becker N. Romeyke S. Schafer S. Domnick F. Spinath F. M. (2015). Intelligence and school grades: a meta-analysis. Intelligence 53, 118–137. 10.1016/j.intell.2015.09.002

[B69] Rushton J. P. Brainerd C. J. Pressley M. (1983). Behavioral development and construct validity: the principle of aggregation. Psychol. Bull. 94, 18–38. 10.1037/0033-2909.94.1.18

[B70] Russell J. (1996). Agency: Its Role in Mental Development. Hove: Taylor & Francis.

[B71] Rust J. (2003). Wechsler Preschool and Primary Scale of Intelligence, (UK Edition), 3rd Edn. London: Harcourt Assessment.

[B72] Rust J. Golombok S. (2005). Wechsler Individual Achievement Test (UK Edition), 2nd Edn. London: Harcourt Assessment.

[B73] Son S. H. Morrison F. J. (2010). The nature and impact of changes in home learning environment on development of language and academic skills in preschool children. Dev. Psychol. 46, 1103–1118. 10.1037/a0020065 20822226

[B74] Sulik M. J. Blair C. Mills-Koonce R. Berry D. Greenberg M. (2015). Early parenting and the development of externalizing behavior problems: longitudinal mediation through children's executive function. Child Dev. 86, 1588–1603. 10.1111/cdev.12386 26082032 PMC4567899

[B75] Totsika V. Sylva K. (2004). The home observation for measurement of the environment revisited. Child Adolesc. Ment. Health 9, 25–35. 10.1046/j.1475-357X.2003.00073.x 32797621

[B76] Trzaskowski M. Harlaar N. Arden R. Krapohl E. Rimfeld K. McMillan A.. (2014). Genetic influences on family socio-economic status and children's intelligence. Intelligence 42, 83–88. 10.1016/j.intell.2013.11.002 24489417 PMC3907681

[B77] Ursache A. Blair C. Raver C. C. (2012). The promotion of self-regulation as a means of enhancing school readiness and early achievement in children at risk for school failure. Child Dev. Perspect. 6, 122–128. 10.1111/j.1750-8606.2011.00209.x PMC7100892 32226480

[B78] Vernon-Feagans L. Willoughby M. Garrett-Peters P. (2016). Predictors of behavioral regulation in Kindergarten: household chaos, parenting and early executive functions. Dev. Psychol. 52, 430–441. 10.1037/dev0000087 26751500 PMC4760868

[B79] Vygotsky L. S. (1978). Mind in Society: The Development of Higher Psychological Processes. Cambridge, MA: Harvard University Press.

[B80] Wiebe S. A. Espy K. A. Charak D. (2008). Using confirmatory factor analysis to understand executive control in preschool children. Dev. Psychol. 44, 575–587. 10.1037/0012-1649.44.2.575 18331145

[B81] Willoughby M. T. Blair C. B. Wirth R. J. Greenberg M. (2012). The measurement of executive function at age 5: psychometric properties and relationship to academic achievement. Psychol. Assess. 24, 226–239. 10.1037/a0025361 21966934 PMC3315058

[B82] Wood D. Bruner J. S. Ross G. (1976). The role of tutoring in problem solving. J. Child Psychol. Psychiatry 17, 89–100. 10.1111/j.1469-7610.1976.tb00381.x 932126

[B83] Wood D. Middleton D. (1975). A study of assisted problem-solving. Br. J. Psychol. 66, 181–191.

[B84] Wood D. Wood H. (1996). Vygotsky, tutoring and learning. Oxford Rev. Educ. 22, 5–16. 10.1080/0305498960220101

[B85] Zelazo P. D. (2006). The Dimensional Change Card Sort (DCCS): a method of assessing executive function in children. Nat. Protoc. 1, 297–301. 10.1038/nprot.2006.46 17406248

